# Inhibition of M/K_v_7 Currents Contributes to Chloroquine-Induced Itch in Mice

**DOI:** 10.3389/fnmol.2020.00105

**Published:** 2020-06-30

**Authors:** Dong Zhang, Hongchao Men, Ludi Zhang, Xiangxin Gao, Jingjing Wang, Leying Li, Qiaoying Zhu, Hailin Zhang, Zhanfeng Jia

**Affiliations:** ^1^Department of Pharmacology, Hebei Medical University, Shijiazhuang, China; ^2^Center for Innovative Drug Research and Evaluation, Institute of Medical Science and Health, Hebei Medical University, Shijiazhuang, China; ^3^The Key Laboratory of Neural and Vascular Biology, Ministry of Education, Shijiazhuang, China; ^4^The Key Laboratory of New Drug Pharmacology and Toxicology, Shijiazhuang, China; ^5^Department of Anesthesiology, Hebei General Hospital, Shijiazhuang, China

**Keywords:** M/K_v_7 potassium currents, dorsal root ganglion neurons, inhibition, chloroquine, itch

## Abstract

M/K_v_7 potassium channels play a key role in regulation of neuronal excitability. Modulation of neuronal excitability of primary sensory neurons determines the itch sensation induced by a variety of itch-causing substances including chloroquine (CQ). In the present study, we demonstrate that suppression of M/K_v_7 channel activity contributes to generation of itch in mice. CQ enhances excitability of the primary sensory neurons through inhibiting M/K_v_7 potassium currents in a Ca^2+^ influx-dependent manner. Specific M/K_v_7 channel opener retigabine (RTG) or tannic acid (TA) not only reverses the CQ-induced enhancement of neuronal excitability but also suppresses the CQ-induced itch behavior. Systemic application of RTG or TA also significantly inhibits the itch behavior induced by a variety of pruritogens. Taken together, our findings provide novel insight into the molecular basis of CQ-induced itch sensation in mammals that can be applied to the development of strategies to mitigate itch behavior.

## Introduction

M/K_v_7 channels are extensively distributed in central and peripheral nerve system (Brown and Passmore, 2009) and play a crucial role in the stabilization of membrane potential and modulation of neuronal excitability (Jentsch, 2000). For example, M/K_v_7 channels determine the resting membrane potential (RMP) of dorsal root ganglion (DRG) neurons (Du et al., 2014) and the firing patterns of action potentials (APs) in superior cervical ganglion (SCG) sympathetic neurons (Jia et al., 2008); loss-of-function mutations of M/K_v_7 channel subunits located in the brain result in epilepsy (Biervert et al., 1998; Singh et al., 1998; Allen et al., 2014).

Importance of M/K_v_7 channels have been demonstrated in a variety of pathological conditions. In the brain, inhibition of M/K_v_7 channels by M1 muscarinic receptor in basolateral amygdala promotes fear memory consolidation (Young and Thomas, 2014); augmentation of M/K_v_7 channels reduces cerebral ischemic stroke-induced brain injury (Bierbower et al., 2015). In the peripheral nerve system, inhibition of M/K_v_7 currents of DRG neurons contributes to acute inflammatory pain induced by pro-inflammatory factors such as proteases (Linley et al., 2008) and bradykinin (Liu et al., 2010); down-regulation of M/K_v_7 expression in DRG neurons mediates chronic inflammatory pain (Mucha et al., 2010), neuropathic pain (Rose et al., 2011; King et al., 2014; Zhang et al., 2019), and cancer pain (Zheng et al., 2013). Activation of peripheral M/K_v_7 channels significantly relives gout pain (Zheng et al., 2015). Thus, suppression of neuronal excitability via augmentation of peripheral M/K_v_7 currents is a valuable analgesic strategy.

Itch, also known as pruritus, is an uncomfortable everyday experience that evokes a desire to scratch (Dong and Dong, 2018). Itch is commonly caused by chemical pruritogens (Dong and Dong, 2018); meanwhile, itch is also a prominent symptom of many diseases, such as dry skin (Pereira and Ständer, 2018), psoriasis (Elewski et al., 2019), and atopic/allergic dermatitis (Bieber, 2008), as well as patients with system disease such as chronic cholestatic liver disease (Carey et al., 2015) and renal disease (Simonsen et al., 2017). Understanding the neural basis of itch at the molecular, cellular, and circuit levels can identify new therapeutic targets to treat this devastating symptom (Meixiong and Dong, 2017). Mammals appear to have evolved two forms of itch: (i) chemical itch, which is activated by chemical mediators such as histamine (HIS) and chloroquine (CQ) and can be effectively gated by noxious painful stimuli (Han and Dong, 2014), and (ii) mechanical itch, which is evoked by light mechanical stimuli such as when insects or parasites come in contact with the skin (Bourane et al., 2015; Dong and Dong, 2018). Mechanical itch transmission is gated by a population of spinal inhibitory interneurons that expressed neuropeptide Y (NPY) (Bourane et al., 2015). Loss of Merkel cells and associated mechanosensitive Piezo2 channels in the skin alleviates the activity of these inhibitory NPY interneurons and leads to mechanical itch (Feng et al., 2018). TRP channels such as TRPV1 and TRPA1 expressed in peripheral DRG pruritic neurons can be activated by G-protein-coupled pruriceptors (i.e., HIS-H1 receptor/H4 receptor, Serotonin-H7 receptor, CQ-MrgprA3 receptor), hence initiate itch sensation (Dong and Dong, 2018).

The fundamental step for different sensation generation including pain and itch is that irritants excite sensory neurons through the corresponding receptors and/or ion channels. Given the crucial role of M/K_v_7 currents in controlling neuronal excitability of sensory neurons, we hypothesize that M/K_v_7 may be involved in pruritogen-induced itch sensation. In the present study, itch-like behavior induced by several pruritogens such as CQ, serotonin (5-hydroxytryptamine, 5-HT), capsaicin (CAP), HIS, carvacrol (CAR), and β-alanine (β-ALA) have been measured. We demonstrate that CQ acts as a novel inhibitor of M/K_v_7 currents and its inhibition of M/K_v_7 currents at least partially contributes to CQ-induced itch in mice.

## Materials and Methods

### Animals

Male C57B/J mice (6–8 weeks old; 20–25 g) were used. Mice were purchased from Beijing Vital River Laboratory Animal Technology Co., Ltd. (Beijing, China) and housed with a stable humidity (55 ± 15%) with free access to food/water in a 12/12 h light–dark cycle at room temperature (22–24°C), according to the guidelines of the local Animal Care and Use Committee at Hebei Medical University (Shijiazhuang, China).

### Chemicals and Antibodies

All chemicals except β-ALA were purchased from Sigma (Sigma-Aldrich LLC, St. Louis, MO, United States). β-ALA was purchased from Beijing Brinway Technology Co. (Beijing, China). Antibodies specific to mouse K_v_7.2 subunit and mouse MrgprA were purchased from Abcam (Ambridge, United Kingdom). Goat anti-rabbit (Fluorescein 5-isothiocyanate, FITC) and Goat anti-mouse (Tetramethylrhodamine, TRITC) were purchased from Jackson ImmunoResearch Inc. (Baltimore, United States). 4′,6-Diamidino-2-phenylindole (DAPI) was purchased from Protein Tech Group, Inc. (Wu Han, China).

### Itch Behavior Test

The itch-like behavior test was performed as previously described (Shimada and LaMotte, 2008; Wilson et al., 2011). Briefly, for assessing CQ-evoked itch behavior, mice received a subcutaneous injection into the cheek (20 μl) or neck (50 μl), with 200 μg CQ dissolved in saline (the concentrations were 20 and 8 mM, respectively). Mice were videotaped for 30 min following the injection. The number of scratch bouts were quantified over a 30-min period. One bout of scratching was defined as an episode in which a mouse lifted its hind paw and scratched continuously for any length of time, until the hind paw was returned to the floor or to the mouth. For assessing XE991 (a specific M/K_v_7 channel blocker)-evoked itch-like behaviors, mice received a subcutaneous injection into the cheek (0.1 or 1 mM, 20 μl) or neck (0.1 or 1 mM, 50 μl). Pruritogen such as 5-HT (1 mM, 50 μl)-, CAP (10 μM, 50 μl)-, HIS (90 mM, 50 μl)-, CAR (70 mM, 50 μl)-, and β-ALA (50 mM, 50 μl)-induced itch-like behaviors by subcutaneous injection into the neck were measured, respectively.

### Rota-Rod Test

Rota-rod test was performed as previously described (Chindo et al., 2014) to assess motor coordination in mice. Briefly, mice were trained to remain on a treadmill device (Jixing Rota-Rod, Hebei, China) with slowly revolving rods of 5 cm diameter at 20 rpm for 180 s. Mice that were able to remain on the rod for 180 s or longer were selected and divided into three groups of five mice per group. Group I received saline, while groups II and III received RTG (20 mg/kg) and TA (16 mg/kg) by intraperitoneal injection, respectively. Thirty minutes after the treatment, mice were placed individually on the rod at intervals of 30 min, up to 60 min. If an animal failed more than once to remain on the rod for 3 min, the test was considered positive, meaning there was a lack of motor coordination.

### Immunostaining

Mice were transcardially perfused with 4% PFA under depth of anesthesia (2% sodium pentobarbital, 30 mg/kg). DRGs were removed and stored in 4% PFA followed by embedding in OCT (SAKURA, Japan). Ten-micrometer DRG sections were cut using a freezing microtome (Leica, Germany). Sections were washed once with 0.1 mol/L PBS (Beijing Solarbio Science & Technology Co., Ltd.) and punched for 30 min in 37°C with 0.3% Triton X-100/PBS buffer and blocked for 1 h with blocking buffer (10% goat serum in 0.1 mol/L PBS). Primary antibodies were diluted in 0.1% Triton X-100/PBS buffer before overnight incubation at 4°C. The second day, sections received three additional washes in PBS before incubation with secondary antibodies for 2 h at 37°C. Sections were washed three times with PBS and placed on microscope slides in Vectashield with DAPI (Southern Biotech). Staining was visualized and captured using a laser scanning confocal microscope (Leica SP5, Leica, Germany).

### Cell Culture

Primary cultures of DRG neurons were prepared from 6- to 8-week-old male mice (20–25 g). Briefly, mice were anesthetized with isoflurane and sacrificed by decapitation. DRGs were rapidly dissected out bilaterally and incubated with 0.2% collagenase and 0.5% dispase for 1 h at 37°C in minimum essential medium for suspension culture (Thermo Fisher Scientific, Inc., Waltham, MA, United States). After digestion and trituration to dissociate neurons, DRG neurons were plated on glass coverslips pre-coated with poly-D-lysine (12.5 μg/ml) and laminin (20 μg/ml in Hanks’ buffered salt solution, BD Biosciences). The cells were cultured in minimum essential medium (Thermo Fisher Scientific, Inc., Waltham, MA, United States) that contained 2.5S NGF (10 ng/ml; Roche Applied Science, Indianapolis, IN, United States), 5% heat-inactivated horse serum (JRH Biosciences, Lenexa, KS, United States), uridine/5-fluoro-2′-deoxyuridine (10 μM), 8 mg/ml glucose, and 1% vitamin solution (Thermo Fisher Scientific, Inc., Waltham, MA, United States). The cultures were maintained in an incubator at 37°C with a humidified atmosphere of 95% air + 5% CO_2_. Cells were used for patch clamp recordings after culturing for 2 days.

### Electrophysiology

Whole-cell mode patch recordings in voltage- and current-clamp configurations were performed at room temperature (22–24°C). Coverslips with cultured neurons were placed in a 0.5-ml recording chamber. The recording chamber was mounted on a stage of an Olympus IX71 inverted microscope (Olympus Corporation, Tokyo, Japan) and cells were continuously perfused at 2 ml/min with bath solution. CQ was applied through bath solution perfusion. The bath solution contained (in mM): 145 NaCl, 5 KCl, 2 MgCl_2_, 2 CaCl_2_, 10 glucose, and 10 HEPES, with an osmolarity of 320 mOsm and a pH of 7.35. The intracellular solution contained (in mM): 150 KCl, 2.4 MgCl_2_, 0.5 CaCl_2_, 5 EGTA, 10 HEPES, 5.0 Na_2_ATP, and 0.33 GTP-Tris salt, with a pH of 7.35 and an osmolarity of 320 mOsm. The recording electrodes were fabricated from thin wall borosilicate glass capillaries using a Flaming P-97 puller (Sutter Instrument Co., Novato, CA, United States) and had resistances of 3–5 MΩ. Signals were recorded with an Axonpatch 700B amplifier, filtered at 2 kHz, and sampled at 5 kHz using pCLAMP 10.7 (Axon Instruments; Molecular Devices, LLC, Sunnyvale, CA, United States). The protocol used to study M/K_v_7 currents of DRG neurons was as follows: the cells were held at −20 mV following a 1-s hyperpolarizing step to −60 mV every 4 s (Jia et al., 2008). We used the current-clamp method to record the APs of DRG neurons. Continuous current-clamp recording with no current injection was used for monitoring of membrane potential (Vm). For recording of APs, cells were held at 0 pA and the APs were elicited by current injection at near the twofold rheobase for 0.5 s. The bath solution and internal solution used to record neuronal APs was the same as that used for M/K_v_7 current recordings. Whole-cell patch clamp recording data were analyzed using Clampfit 10.7 software (Axon Instruments; Molecular Devices, LLC, Sunnyvale, CA, United States).

### Calcium Imaging

DRG neurons were loaded with 2 μM fluo-4-acetoxymethyl ester (fluo-4-AM; Molecular Probes; Thermo Fisher Scientific, Inc.) at 37°C for 30 min. After loading, the cells were washed three times with Dulbecco’s PBS to remove the extracellular dye, and then placed in a chamber mounted on the stage of laser scanning confocal microscope (Leica TCS SP5; Leica Microsystems GmbH, Wetzlar, Germany). The cells were incubated with the same bath solution as the patch clamp experiment. Fluo-4-AM loaded calcium signals were excited at a wavelength of 488 nm, and the emission fluorescence was measured at 530 nm. The calcium signals induced by drug application in bath solution were measured. Dynamic signals were recorded at an interval of 2 s and normalized to the initial fluorescence value.

### Statistics

Data were presented as the mean ± SEM for the indicated number of independently conducted experiments, and analyzed with Origin 9.1 software (OriginLab Corporation, Northampton, MA, United States) and SPSS 13.0 (SPSS Inc., Chicago, IL, United States). Statistical significance was evaluated using either a Student’s *t*-test or a one-way analysis of variance followed by Dunnett’s *post-hoc* test for multiple groups. For the behavioral data that were not in normal distribution, Mann–Whitney *U* test was used to evaluate the statistical significance. *p* < 0.05 was considered to indicate a statistically significant difference.

## Results

### Inhibition of M/K_v_7 Channel Activity Induces Itch-Like Behavior in Mice

**FIGURE 1:**
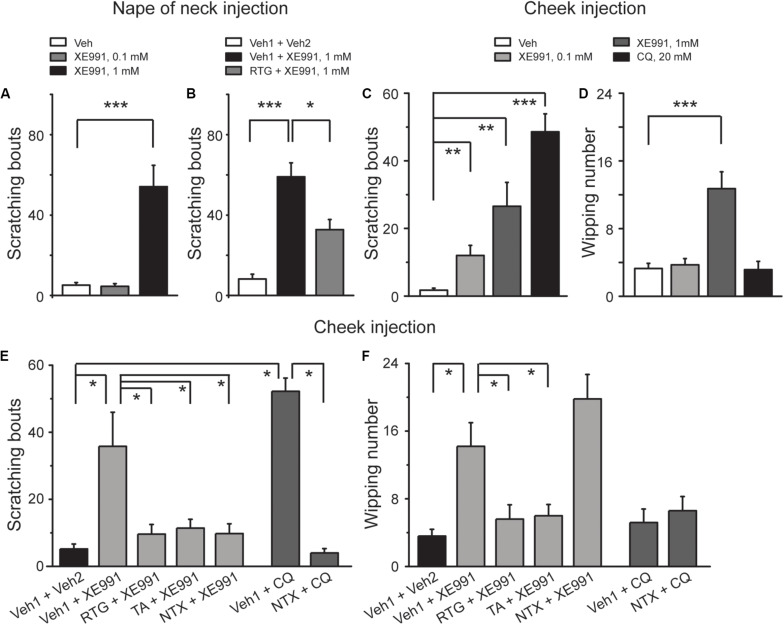
Inhibition of M/K_v_7 channel activity induces itch in mice. **(A)** Intracutaneous injection of XE991 (1 mM, 50 μl) into the nape of neck induces scratching behavior. **(B)** Intracutaneous pre-injection of M/K_v_7 channel opener retigabine (RTG, 10 mg/kg, 100 μl) suppresses XE991-induced scratching behavior. **(C,D)** Intracutaneous injection of XE991 into the cheek induces scratching **(C)** and pain behavior **(D)**, respectively. As a positive control, pruritogen CQ (20 mM, 20 μl) only induces itch **(C)** but not pain behavior **(D)**. **(E,F)** Intracutaneous pre-injection of RTG (15 mM, 25 μl) and tannic acid (TA, 5 mM, 25 μl) significantly suppresses XE991 (1 mM)-induced scratching **(E)** and pain behavior **(F)** in cheek model. Intracutaneous pre-injection of naltrexone (1 mg/kg, 25 μl) only inhibits XE991 (1 mM)-induced scratching **(E)** but not pain behavior **(F)**. As a positive control, CQ (20 mM, 20 μl) only induces itch behavior **(E)**, and it can be reversed by naltrexone. Data are shown as mean ± SEM (*n* = 8 per group in **A–D** and *n* = 5 per group in **E** and **F**). The statistical significance was evaluated using Student’s *t-*test. *p < 0.05, **p < 0.01, ***p < 0.001.

To determine the role of M/K_v_7 channel in the generation of itch sensation, itch-like behavior test *in vivo* was performed in mice. Intracutaneous injection of 1 mM XE991, a selective M/K_v_7 channel blocker (Wang et al., 1998) into the nape of neck, elicited obvious scratching behavior (Figure 1A). The scratching behavior induced by XE991 was significantly suppressed by the specific M/K_v_7 channel opener retigabine (RTG) (Tatulian et al., 2001; Figure 1B). These results suggest that inhibition of M/K_v_7 channel activity induces itch-like behavior in mice.

To distinguish the itch-like behavior from the painful behavior that could also be induced by inhibition of M/K_v_7 channel activity (Liu et al., 2010; Du et al., 2014), agents were intracutaneously injected into the cheek (known as “cheek model of itch”). In this case, the itch-like behavior will be demonstrated by hindpaw scratching, whereas the painful behavior will be demonstrated by forepaw wiping, respectively (Shimada and LaMotte, 2008; Wilson et al., 2011). Indeed, cheek injection of 0.1 and 1 mM XE991 both induced significant scratching behavior (Figure 1C). However, only 1 mM XE991 induced pain behavior (Figure 1D). As a positive control, cheek injection of CQ (20 mM) elicited obvious scratching (Figure 1C) but not pain behavior (Figure 1D). In the cheek model, RTG and another M/K_v_7 channel opener tannic acid (TA) (Zhang et al., 2015) were used to further determine the role of M/K_v_7 channel in itch and pain. XE991 (1 mM)-induced scratching and pain behavior were significantly suppressed by intracutaneous pre-injection of RTG (15 mM) and TA (5 mM) (Figures 1E,F). Naltrexone (1 mg/kg), a well-known itch antagonist (Spradley et al., 2012), significantly inhibited 1 mM XE991-induced scratching but not pain behavior by intracutaneous pre-injection (Figures 1E,F). CQ-induced scratching behavior was also significantly suppressed by intracutaneous pre-injection of naltrexone (1 mg/kg) (Figures 1E,F). On the other hand, rota-rod test was performed to exclude the possibility that M/K_v_7 channel inhibition-induced itch and pain were suppressed by M/K_v_7 channel openers through inhibiting the central nervous system (CNS) and motor function. As shown in Table 1, intraperitoneal injection of RTG (20 mg/kg) or TA (16 mg/kg) did not affect CNS and motor function at the time points when the itch behavior tests were performed (Table 1). Taken together, these results indicate that inhibition of M/K_v_7 channel activity contributes to not only pain but also itch sensation generation.

**TABLE 1:** Effects of intraperitoneal injection of RTG and TA on rota-rod test in mice.

Treatment	Post-treatment (min)			
	30		60	
	Time on rod %	Fail.%	Time on rod %	Fail.%
Saline	>180.0 s	0.0	>180.0 s	0.0
RTG 20 mg/kg	>180.0 s	0.0	>180.0 s	0.0
TA 16 mg/kg	>180.0 s	0.0	>180.0 s	0.0

*Agents were administrated by intraperitoneal (i.p.) injection. Rota-rod test was performed after treatment for 30 and 60 min, respectively. Thirty minutes was used because it was the start time to observe the effects of CQ after i.p. administration of RTG or TA in the experiments. Results are presented as mean ± SEM. No significant difference between saline and treated groups; one-way analysis of variance (ANOVA) followed by Dunnett’s post-hoc test for multiple comparisons; n = 5 in each group.*

### M/K_v_7 Channel Openers Selectively Suppress CQ-induced Itch-Like Behavior in Mice

**FIGURE 2:**
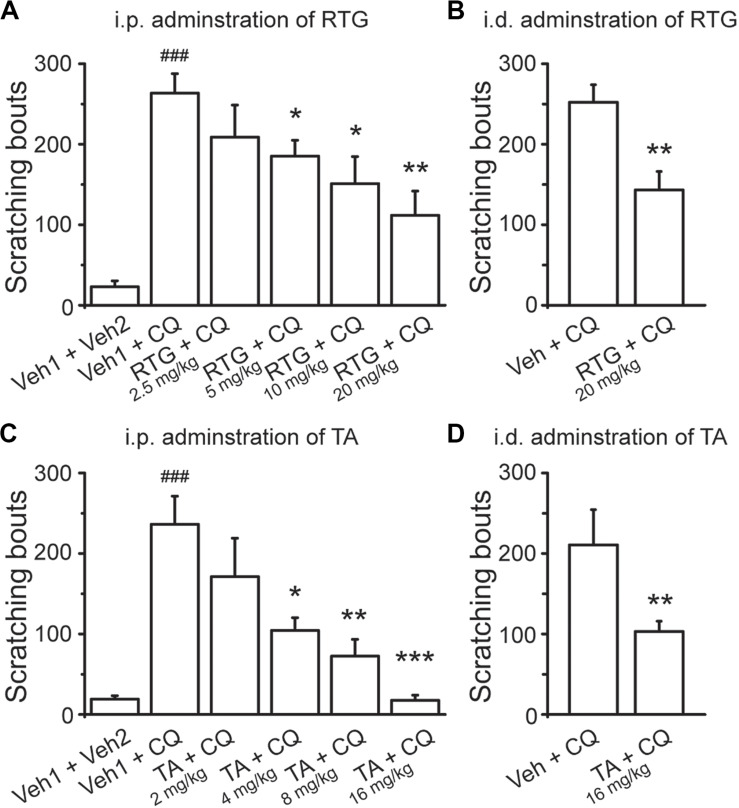
Activation of M/K_v_7 channel suppresses CQ-evoked itch. Intraperitoneal (i.p.) pre-injection of RTG **(A)** and TA **(C)** suppresses CQ (8 mM, 50 μl)-evoked scratching behavior in a dose-dependent manner. Intradermal (i.d.) pre-injection of RTG (20 mg/kg, 100 μl) **(B)** and TA (16 mg/kg, 100 μl) **(D)** reduces the same site injection of CQ (8 mM, 50 μl)-evoked scratching behavior. Data are shown as mean ± SEM (*n* = 8 per group). The statistical significance was evaluated using one-way analysis of variance followed by Dunnett’s *post-hoc* test for comparation among multiple groups and Student’s *t-*test between two groups. *p < 0.05, **p < 0.01, ***^/###^*p* < 0.001.

To determine the role of M/K_v_7 channel in CQ-induced itch sensation, the *in vivo* behavioral test was performed in mice whose nape of neck was intradermally injected with CQ; the itch sensation was identified by the scratching on the skin with hindpaws. RTG and TA were tested in the scratching behavioral measurement. Intradermal injection of CQ significantly increased the scratching bouts to 263 ± 24 (*n* = 8) from 23 ± 8 (*n* = 8, *p* < 0.001) in control group mice (Figure 2A). Intraperitoneal pre-administration of RTG or TA effectively prevented CQ-induced scratching behavior in a dose-dependent manner (Figures 2A,C). For example, pre-administration of RTG at 5, 10, and 20 mg/kg significantly reduced the scratching bouts of CQ group from 263 ± 24 to 185 ± 20 (*n* = 8, *p* < 0.05), 151 ± 33 (*n* = 8, *p* < 0.05), and 112 ± 30 (*n* = 8, *p* < 0.01), respectively (Figure 2A). Pre-administration of TA at 4, 8, and 16 mg/kg also significantly reduced CQ-induced scratching bouts from 236 ± 35 (*n* = 8) to 104 ± 16 (*n* = 8, *p* < 0.05), 73 ± 21 (*n* = 8, *p* < 0.01), and 18 ± 6 (*n* = 8, *p* < 0.001), respectively (Figure 2C). Furthermore, intradermal pre-injection of RTG and TA at the nape of neck significantly reduced the scratching bouts induced by CQ injected at the same site from 252 ± 22 (*n* = 8) and 211 ± 44 (*n* = 8) to 143 ± 23 (*n* = 8, *p* < 0.01) and 103 ± 13 (*n* = 8, *p* < 0.01), respectively (Figures 2B,D). These results indicate that the M/K_v_7 channel is involved in the CQ-induced itch sensation.

**FIGURE 3:**
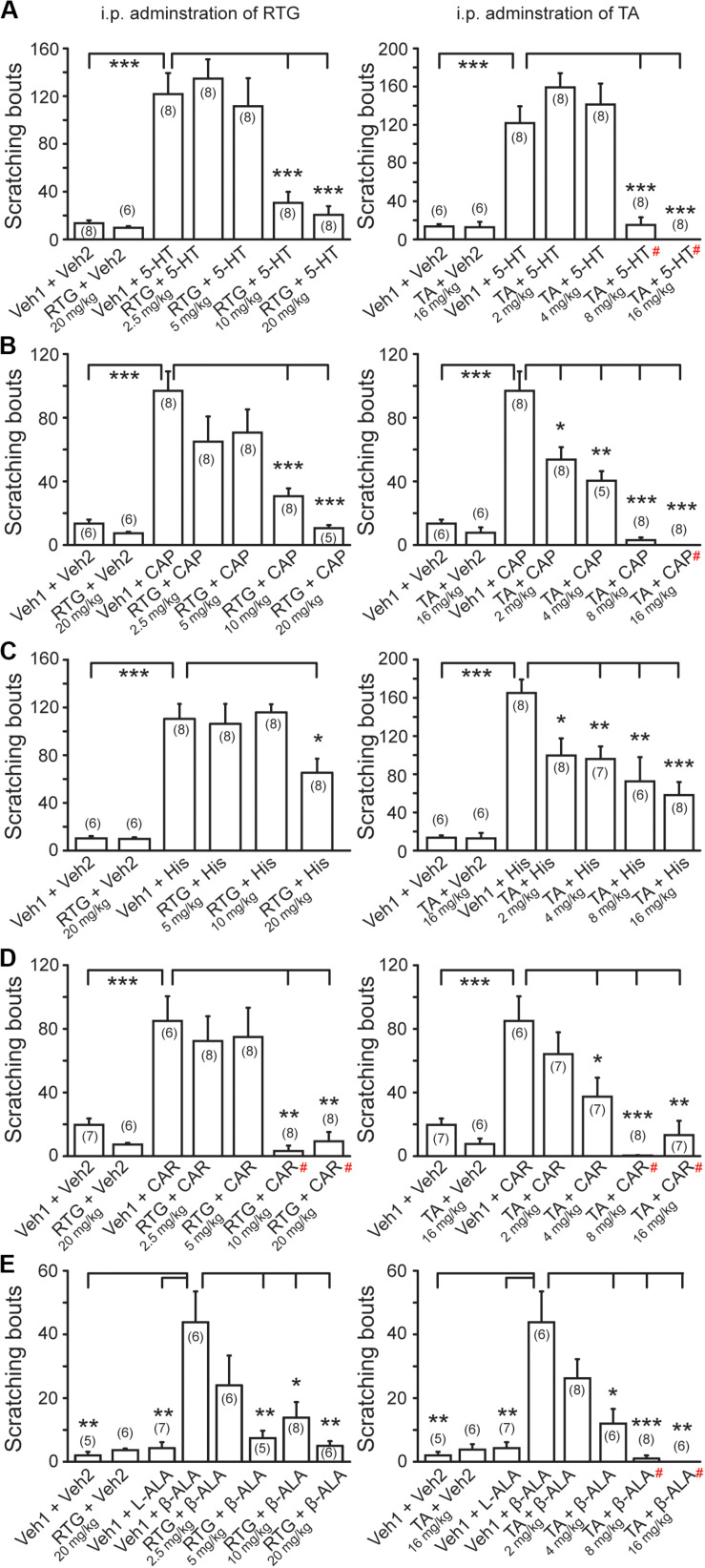
Effects of M/K_v_7 channel activation in several pruritogens-evoked itch. I.p. pre-injection of RTG (left) and TA (right) suppresses serotonin (5-HT, 1 mM, 50 μl) **(A)**, capsaicin (CAP, 10 μM, 50 μl) **(B)**, histamine (His, 90 mM, 50 μl) **(C)**, carvacrol (CAR, 70 mM, 50 μl) **(D)**, and β-alanine (β-ALA, 50 mM, 50 μl) **(E)**-evoked scratching behavior in a dose-dependent manner, respectively. Data are shown as mean ± SEM (the *n* number in each group is indicated). For the data that exhibited normal distribution, the statistical significance was evaluated using Student’s *t-*test between two groups; for the data that were not in normal distribution (#), the statistical significance was evaluated using Mann–Whitney *U* test between two groups. *p < 0.05, **p < 0.01, ***p < 0.001.

We then examined whether the M/K_v_7 channel is also involved in itch sensation induced by other pruritogens. For this, 5-HT, CAP, HIS, CAR, and β-ALA were intradermally injected into the nape of neck, respectively. All the above compounds elicited obvious itch-like scratching behavior (Figures 3A–E). Intraperitoneal pre-administration of RTG or TA significantly prevented this compound-induced scratching behavior in a dose-dependent manner (Figures 3A–E). Taken together, these results indicate that augmentation of M/K_v_7 channel activity significantly suppresses the itch sensation induced by the above compounds.

### CQ Inhibits M/K_v_7 Currents Through Increasing Intracellular Ca^2+^ in Mouse DRG Neurons

**FIGURE 4:**
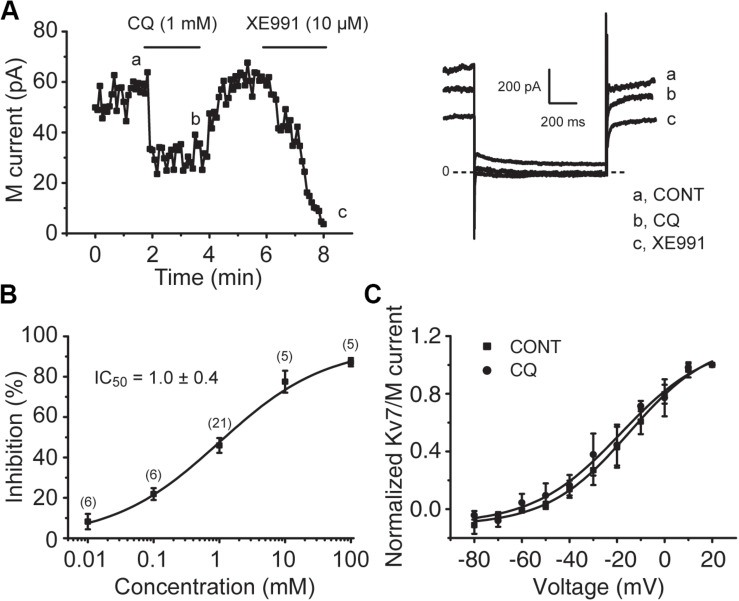
CQ inhibits M/K_v_7 currents in mice DRG neurons. **(A)** Representative time course of CQ (1 mM)-induced inhibition of M/K_v_7 currents (left). Current traces are shown (right). XE991 (10 μM) was used as positive control to inhibit M/K_v_7 currents. **(B)** The concentration–effect relationship curve for CQ-induced inhibition of M/K_v_7 currents is shown. IC_50_ = 1.0 ± 0.4 mM (*n* = 6, 6, 21, 5, 5 at each concentration of CQ, respectively). **(C)** CQ (1 mM) has no significant effect on current–voltage (*I–V*) curve of M/K_v_7 channel. The statistical significance was evaluated using one-way analysis of variance followed by Dunnett’s *post-hoc* test for multiple comparations.

To determine the effect of CQ on M/K_v_7 currents, whole-cell patch-clamp recordings were performed in cultured mouse DRG neurons. Bath application of CQ significantly inhibited M/K_v_7 currents in a concentration-dependent manner (Figures 4A,B). CQ initiated its inhibition on M/K_v_7 currents at 0.01 mM and reached its maximal inhibition at 100 mM (Figure 4B). The half maximum inhibitory concentration (IC_50_) was at 1.0 ± 0.4 mM (Figure 4B). However, the current–voltage (*I*–*V*) curve of M/K_v_7 was not affected by CQ (Figure 4C). In addition, immunostaining assay showed that M/K_v_7 channel subunit K_v_7.2 was co-localized with MrgprA receptor (CQ receptor) in mouse DRG neurons (Supplementary Figure 1).

**FIGURE 5:**
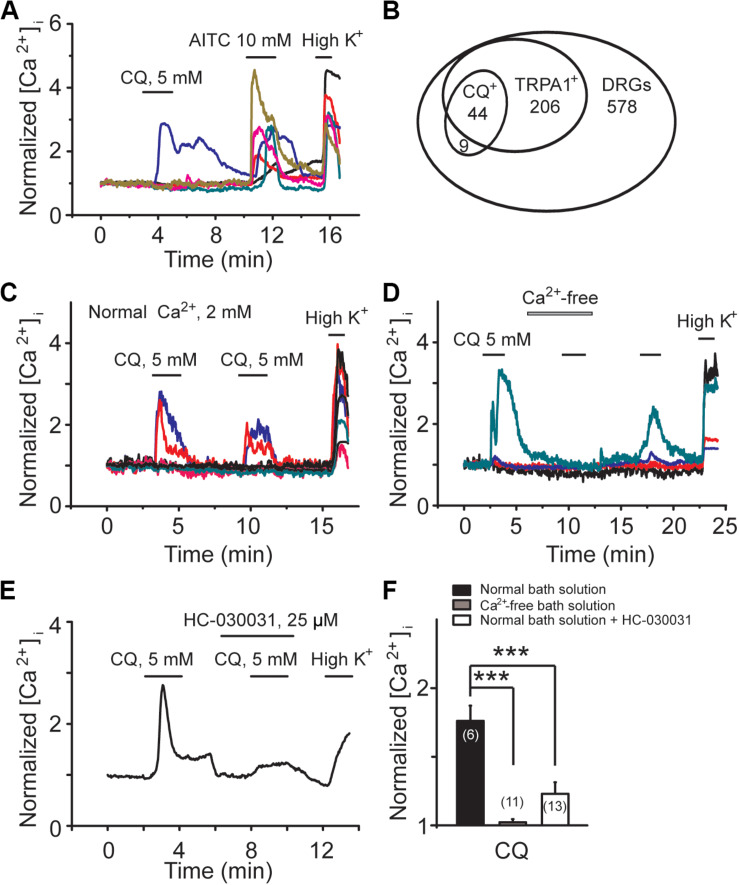
CQ increases intracellular Ca^2+^ through TRPA1-mediated Ca^2+^ influx in mice DRG neurons. **(A)** Representative traces from six different DRG neurons with calcium-imaging assay. Allyl isothiocyanate (AITC, 10 mM)-responsive DRG neurons indicate TRPA1-positive neurons. **(B)** The Venn diagram illustrates the relationships of CQ-, AITC (TRPA1^+^)-responsive neurons in adult DRG. The sizes of the circles are proportional to the sizes of the cell populations. CQ can increase intracellular Ca^2+^ in the presence **(C)** but not in the absence **(D)** of extracellular Ca^2+^ (2 mM). **(E)** Selective inhibition of TRPA1 by HC-030031 reduces CQ-induced increase of intracellular Ca^2+^. **(F)** Summary data of CQ-induced increase of intracellular Ca^2+^ in normal bath solution, Ca^2+^-free solution, and normal bath solution contained HC-030031, respectively. Data are shown as mean ± SEM (the *n* number in each group is indicated). The statistical significance was evaluated using Student’s *t-*test for comparison between two groups. ***p < 0.001.

For the signaling molecules involved in M/K_v_7 current inhibition, three mechanisms have been proposed, PIP_2_ depletion, increment of intracellular Ca^2+^, and PKC phosphorylation, which are the downstream signals of phospholipase C (PLC) activation (Gamper and Shapiro, 2015; Du et al., 2018). Previous studies have demonstrated that CQ increases intracellular concentration of Ca^2+^ through activation of TRPA1 channels (Wilson et al., 2011; Than et al., 2013). We examined the effect of CQ on mouse DRG neurons using Ca^2+^-imaging assay. Specific TRPA1 agonist allyl isothiocyanate (AITC) was used to indicate the DRG neurons with TRPA1-positive response. As shown in Figure 5, CQ significantly increased intracellular Ca^2+^ in mouse DRG neurons (Figure 5A). Eighty-three percent (44 out of 53) of mouse DRG neurons that responded to CQ were TRPA1-positive DRG neurons (Figures 5A,B). Excluding the Ca^2+^ from the bath solution eliminated CQ-induced increase of intracellular Ca^2+^ (Figures 5C,D,F). HC-030031, a specific TRPA1 channel blocker, significantly inhibited CQ-induced increase of intracellular Ca^2+^ (Figures 5E,F). These results indicate that CQ-induced intracellular Ca^2+^ increase in mouse DRG neurons is due to the TRPA1 activation-induced Ca^2+^ influx.

**FIGURE 6:**
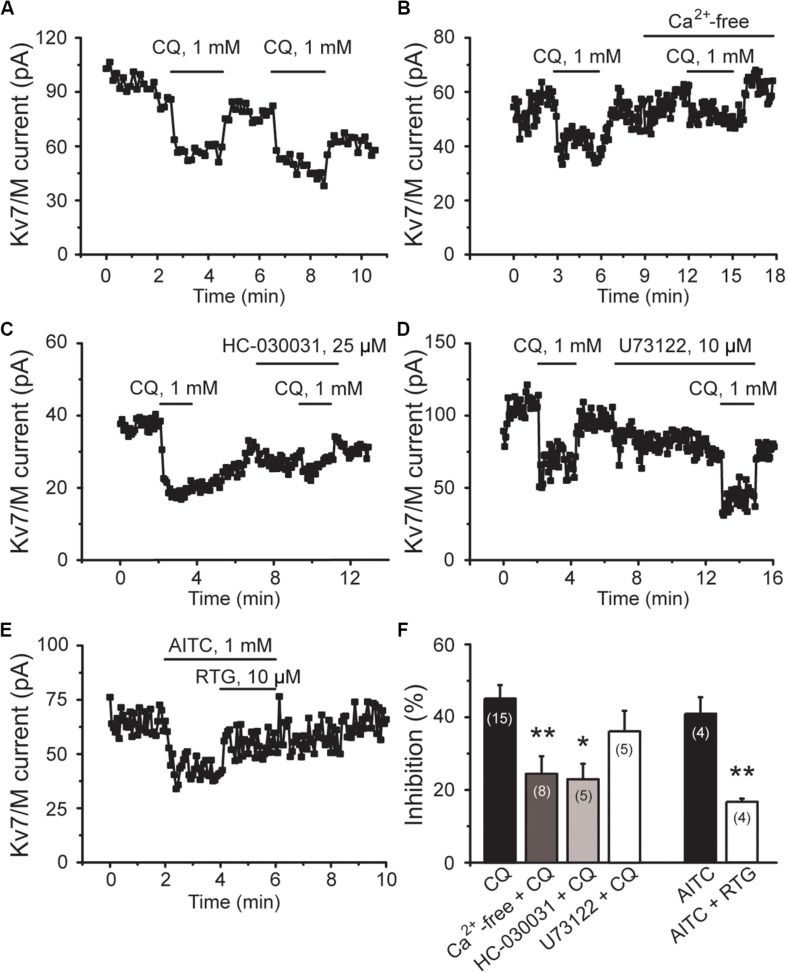
CQ inhibits M/K_v_7 currents through TRPA1-mediated Ca^2+^ influx in mice DRG neurons. **(A)** Representative time course of CQ (1 mM)-induced inhibition of M/K_v_7 currents. **(B)** Excluding the Ca^2+^ from the bath solution eliminates CQ-induced inhibition of M/K_v_7 currents. **(C)** Selective inhibition of TRPA1 by HC-030031 suppresses CQ-induced inhibition of M/K_v_7 currents. **(D)** Inhibition of phospholipase C by U73122 does not suppress CQ-induced inhibition of M/K_v_7 currents. **(E)** Selective activation of TRPA1 by AITC inhibits M/K_v_7 currents, and this effect can be reduced by RTG. **(F)** Summary data from **(A–E)**. Data are shown as mean ± SEM (the *n* number in each group is indicated). The statistical significance was evaluated using Student’s *t-*test for comparison between two groups. *p < 0.05, **p < 0.01.

Consistently, CQ-induced inhibition of M/K_v_7 currents was significantly attenuated by perfusing cells with Ca^2+^-free bath solution and HC-030031, respectively (Figures 6A–C,F). For example, Ca^2+^-free bath solution significantly reduced the CQ (1 mM)-induced inhibition of M/K_v_7 current from 45% (*n* = 15) to 24% (*n* = 8, *p* < 0.01), while HC-030031 significantly reduced the inhibition to 23% (*n* = 5, *p* < 0.05) (Figure 6F). However, bath application of U73122, a PLC inhibitor, showed no obvious effects on CQ-induced inhibition of M/K_v_7 currents (36%; *n* = 5, *p* > 0.05) (Figures 6D,F). Furthermore, activation of TRPA1 with AITC (1 mM) significantly inhibited M/K_v_7 currents by 41% (*n* = 4), and this effect was reduced to 17% (*n* = 4, *p* < 0.01) by 10 μM RTG (Figures 6E,F). Taken together, these results suggest that CQ-induced inhibition of M/K_v_7 currents in mouse DRG neurons is due to the CQ-induced increase of intracellular Ca^2+^, which is through TRPA1 activation-mediated Ca^2+^ influx. PIP_2_ depletion and PKC phosphorylation, the downstream signals of PLC, may not be involved in CQ-mediated inhibition of M/K_v_7 currents.

### CQ Enhances the Excitability of Mouse DRG Neurons

**FIGURE 7:**
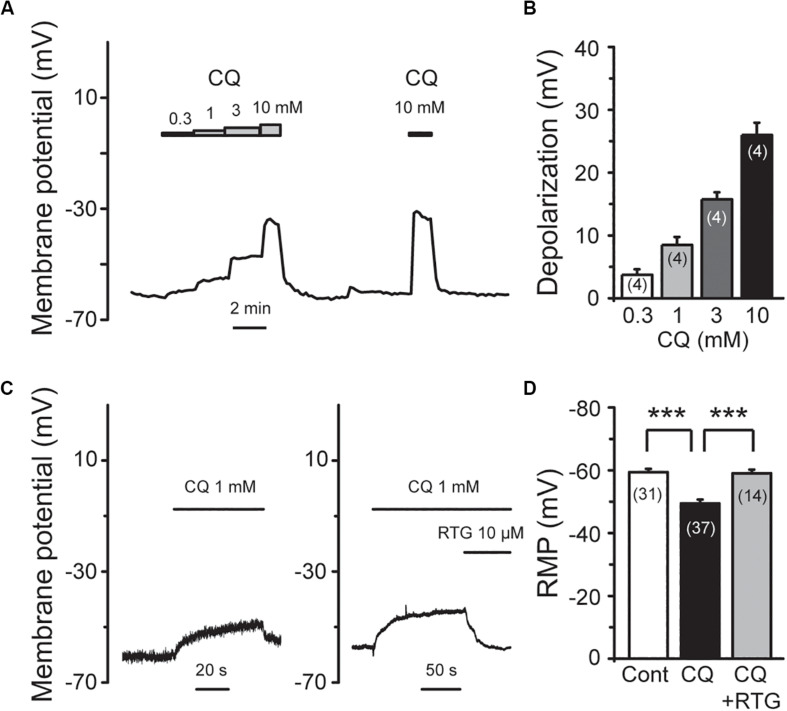
RTG reverses CQ-induced depolarization of the membrane potential in DRG neurons. **(A)** Representative trace shows that CQ depolarizes membrane potential (Vm) in a concentration-dependent manner. **(B)** Summary data of depolarization of membrane potential by different concentration of CQ. **(C)** RTG reverses CQ (1 mM)-induced depolarization of membrane potential. **(D)** Summary data of resting membrane potential (RMP). Data are shown as mean ± SEM (the *n* number in each group is indicated). The statistical significance was evaluated using Student’s *t-*test for comparison between two groups. ***p < 0.001.

**FIGURE 8:**
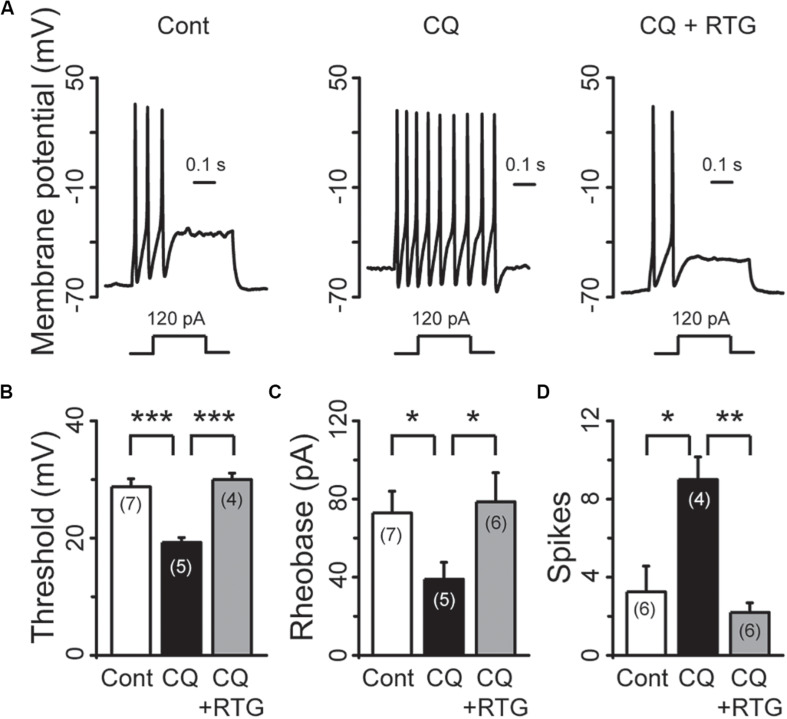
RTG reverses CQ-induced hyperexcitability of DRG neurons. **(A)** Representative samples show that CQ induces hyperexcitability (middle) compared with control (Cont, left), while RTG reverses CQ-induced the neuronal hyperexcitability (right). **(B)** RTG increases CQ (1 mM)-induced reduction of the threshold (*n* = 7, Cont group; *n* = 5, CQ group; and *n* = 4, CQ + RTG group). **(C)** RTG increases CQ (1 mM)-induced reduction of the rheobase. *n* = 7, 5, and 6 in Cont, CQ, and CQ + RTG group, respectively. **(D)** RTG reduces CQ (1 mM)-induced increase of action potentials (spikes) number. *n* = 6, 4, and 6 in Cont, CQ, and CQ + RTG group, respectively. Data are shown as mean ± SEM (the *n* number in each group is indicated). The statistical significance was evaluated using Student’s *t-*test for comparison between two groups. *p < 0.05, **p < 0.01, ***p < 0.001.

M/K_v_7 currents play a key role in regulation of neuronal excitability. As a result, inhibition of M/K_v_7 currents causes membrane depolarization and reduces the threshold and rheobase of action potentials (Du et al., 2018). We next tested whether CQ increased the excitability of mouse DRG neurons through inhibition of M/K_v_7 currents as demonstrated above. The membrane potential of mouse DRG neurons was first recorded using the current clamp method (Figures 7A–C). CQ depolarized the membrane potential in a concentration-dependent manner (Figures 7A,B). At 1 mM, CQ significantly depolarized the membrane potential from −59 ± 1 mV (*n* = 31) to −49.5 ± 1.2 mV (*n* = 37, *p* < 0.001) (Figures 7C,D). Furthermore, the CQ-induced depolarization of membrane potential could be reversed (to 59 ± 1.2 mV, *n* = 14, *p* < 0.001) by the M/K_v_7 channel opener RTG (Figures 7C,D). Then, the effect of CQ on action potentials (APs) of mouse DRG neurons was tested; APs were induced by different levels of current injection. For this, the threshold (the distance from resting membrane potential to threshold membrane potential) and the rheobase for eliciting APs and AP fire numbers was addressed. The threshold was significantly reduced from 28.8 ± 1.4 mV (*n* = 7) of control group to 19.3 ± 0.9 mV (*n* = 5, *p* < 0.001) by CQ (Figures 8A,B), which was mostly reversed by RTG (30 ± 1.1mV, *n* = 4, *p* < 0.001) (Figures 8A,B). Similarly, CQ reduced the rheobase from 73 ± 11 pA (*n* = 7) to 39 ± 9 pA (*n* = 5, *p* < 0.05), and RTG reversed this CQ-induced reduction of the rheobase (to 79 ± 15 pA, *n* = 6, *p* < 0.05) (Figure 8C). Finally, numbers of APs induced by a 120-pA current injection were increased from 3.3 ± 1.3 (*n* = 6) to 9.0 ± 1.2 by CQ (*n* = 4, *p* < 0.05) (Figures 8A,D), which was again reversed by RTG (to 2.2 ± 0.5, *n* = 6, *p* < 0.01) (Figures 8A,D). These results indicate that CQ enhances the excitability of mouse DRG neurons through its inhibition of M/K_v_7 currents.

## Discussion

For chemical itch, a better understanding of peripheral mechanisms is emerging (Dong and Dong, 2018), and to this we add a new mechanism of M/K_v_7 channels. The major findings of this study are as follows: (1) inhibition of M/K_v_7 potassium currents causes itch sensation; (2) CQ is an inhibitor of M/K_v_7 currents, and inhibitory mechanism involves TRPA1-mediated Ca^2+^ influx; (3) CQ-induced inhibition of M/K_v_7 induces hyper-excitability of DRG neurons, which is likely the mechanism for CQ-induced itch sensation. It is well-known that peripheral M/K_v_7 channels play a key role in pain sensation. Augmentation of its expression or activity is an effective analgesic strategy (Du et al., 2018). Interestingly, we found in this study that inhibition of M/K_v_7 also leads to itch generation under the “cheek model of itch” and “neck model of itch” measurements, and activators of M/K_v_7 channels alleviate itch behavior caused by CQ and other pruritogens. This is the first time, to our best knowledge, to describe the relationship between M/K_v_7 channel and itch. In the peripheral sensory system, increasing the expression and/or the activity of M/K_v_7 channels may have great benefit for anti-pruritus.

Among the pruritogens we tested in this study, CQ is a drug that has long been used in the treatment and prevention of malaria. One major side effect of CQ is HIS-independent itch (Ezeamuzie et al., 1990; Abila et al., 1994; Green et al., 2006). Recently, the molecular mechanisms for CQ-induced itch has been studied. One subpopulation of dorsal horn neurons in the spinal cord that specifically expresses gastrin-releasing peptide receptor (GRPR) mediates CQ- and other chemical-induced itch (Sun and Chen, 2007). In the primary DRG sensory neurons, mouse MrgprA3 and human MrgprX1 are functioned as the predominant receptors for CQ (Liu et al., 2009). Mrgprs (known as the Mas-related G protein-coupled receptors) are a family of orphan GPCRs that are restricted to express in the subsets of small-diameter sensory neurons in DRG (Dong et al., 2001). Since all of the molecular correlates of M/K_v_7 channel, KCNQ2/3/5, were widely expressed in DRG neurons including small- and large-diameter sensory neurons (Passmore et al., 2003; Du et al., 2018), it will be not surprised to find that M/K_v_7 channels are co-localized with CQ receptors. Indeed, our results show that MrgprA receptors are co-localized with K_v_7.2 subunit in mouse DRG neurons. CQ activates TRPA1 channel through the activation of the MrgprA3-G_βγ_ pathway, which evokes itch (Wilson et al., 2011). Consistent with the previous study, our results demonstrate that CQ increases intracellular Ca^2+^ concentration by Ca^2+^ influx in a TRPA1-dependent manner in mouse DRG neurons. Furthermore, it is likely that CQ uses this mechanism but not MrgprA3/PLC pathway to inhibit M/K_v_7 currents, because removing extracellular Ca^2+^ or blocking TRPA1 channel both significantly suppresses the CQ-induced inhibition of M/K_v_7 currents, whereas blocking PLC activity does not affect CQ-mediated inhibition of M/K_v_7 currents. Interestingly, TRPA1 activation is likely sufficient to inhibit M/K_v_7 currents directly. It is well-known that increase of intracellular Ca^2+^ induces M/K_v_7 inhibition (Cruzblanca et al., 1998; Gamper and Shapiro, 2003; Kosenko and Hoshi, 2013). Consistent with our observation, TRPV1-mediated Ca^2+^ influx has been shown to inhibit M/K_v_7 currents (Zhang et al., 2011). It is worth noting that CQ still inhibited M/K_v_7 currents in Ca^2+^-free bath solution or in the presence of TRPA1 blocker. This phenomenon indicates that CQ may exhibit non-specific, direct inhibition on M/K_v_7 currents in mouse DRG neurons. Similar to CQ, 5-HT activates TRPA1 channels but through HT receptor 7 (HTR7)–Adenylyl Cyclase (AC)–cAMP pathway that evokes itch sensation (Morita et al., 2015). Genetic knockout of HTR7 completely abolished low dose of 5-HT-induced itch and partially reduced scratching in mouse model of atopic dermatitis (Morita et al., 2015). Thus, both CQ and 5-HT target to activate TRPA1 channel and thereby enhance neuronal excitability to evoke itch sensation (Wilson et al., 2011; Morita et al., 2015). However, whether 5-HT/HTR7/TRPA1-mediated Ca^2+^ influx inhibits M/K_v_7 currents and then contributes to 5-HT-induced itch remains to be determined.

HIS-induced itch sensation is due to exciting sensory neurons through the activation of H1 or H4 receptor (Dunford et al., 2007; Simons and Simons, 2011). Direct activation of TRPV1 channel by HIS-H1R underlies HIS induced itch (Shim et al., 2007). HIS also inhibits M/K_v_7 currents through H1R–PLC pathway-induced membrane PI(4,5)P_2_ hydrolysis in rat SCG neurons (Liu et al., 2008). Moreover, TRPV1 channel-mediated Ca^2+^ influx inhibits M/K_v_7 currents (Zhang et al., 2011). Thus, involvement of M/K_v_7 inhibition in HIS-induced itch sensation is a logical explanation. TRPV1, when activated by its agonist CAP (Caterina et al., 1997, 2000), also evokes moderate itch (Sikand et al., 2009, 2011), besides its being well-known as a nociceptive receptor (Caterina et al., 1997, 2000).

β-ALA is reported to inhibit M/K_v_7 currents through MrgprD receptor in rat DRG neurons (Crozier et al., 2007); also, this may not be the case in mice DRG neurons (Rau et al., 2009). Indeed, β-ALA can evoke HIS-independent itch sensation and increase intracellular Ca^2+^ concentration, both through MrgprD receptor in mice, but whether M/K_v_7 channels are involved remains unclear (Liu et al., 2012). Nonetheless, we show in this study that activation of M/K_v_7 channels also alleviates the β-ALA-induced itch behavior.

CAR is a natural compound that can specifically activate TRPV3 channels (Nilius and Szallasi, 2014; Wang and Wang, 2017). TRPV3 is most abundantly expressed in skin keratinocytes and in cells surrounding hair follicles, where it plays an essential role in cutaneous sensation including thermal sensation, nociception, and itch, in addition to maintenance of the skin barrier and hair growth (Peier et al., 2002; Cheng et al., 2010; Cui et al., 2018). In human, gain-of-function mutations of TRPV3 are associated with Olmsted syndrome, which is characterized by severe itch and palmoplantar and periorificial keratoderma (Lai-Cheong et al., 2012; Lin et al., 2012). In rodents, gain-of-function mutations of TRPV3 are associated with skin inflammation and pruritus (Asakawa et al., 2006; Yoshioka et al., 2009). In addition, itching behavior is suppressed in TRPV3 knockout mice (Yamamoto-Kasai et al., 2012). TRPV3 is unlikely to be distributed in sensory neurons of rodents (Peier et al., 2002). Thus, systemic application of M/K_v_7 opener, RTG, and TA, may indirectly reduce CAR/TRPV3-induced itch by suppressing the excitability of sensory neurons.

In conclusion, the results of this study strongly demonstrate that inhibition of M/K_v_7 currents induces itch. TRPA1-dependent inhibition of M/K_v_7 currents by CQ contributes to CQ-induced itch. These findings provide novel insight into the molecular basis of chemical itch that can be used to develop strategies to mitigate itch sensation.

## Data Availability Statement

All datasets generated for this study are included in the article/Supplementary Material.

## Ethics Statement

The animal study was reviewed and approved by the Animal Care and Use Committee of the Hebei Medical University.

## Author Contributions

HZ and ZJ designed the study and wrote the manuscript. DZ, HM, LZ, XG, JW, LL, and QZ performed the experiments. ZJ, DZ, and HM analyzed the data. All authors contributed to the article and approved the submitted version.

## Conflict of Interest

The authors declare that the research was conducted in the absence of any commercial or financial relationships that could be construed as a potential conflict of interest.

## Supplementary Material

FIGURE S1 Co-localization of K_v_7.2 and MrgprA in mouse DRG neurons. **(A)** Representative images show that M/K_v_7 channel subunit K_v_7.2 is co-localized with MrgprA receptor (arrows, bottom merged) in mouse DRG neurons. Images in the bottom row are extended from the square regions in the up row./break Scale bar in each panel in up and bottom row is 75 and 25 μm, respectively.

## References

[B1] Abila B. Ezeamuzie I. C. Igbigbi P. S. Ambakederemo A. W. Asomugha L. (1994). Effects of two antihistamines on chloroquine and histamine induced weal and flare in healthy African volunteers. *Afr. J. Med. Med. Sci.* 23 139–142. 7625301

[B2] Allen N. M. Mannion M. Conroy J. Lynch S. A. Shahwan A. Lynch B. (2014). The variable phenotypes of KCNQ-related epilepsy. *Epilepsia* 55 e99–e105. 10.1111/epi.12715 25052858

[B3] Asakawa M. Yoshioka T. Matsutani T. Hikita I. Suzuki M. Oshima I. (2006). Association of a mutation in TRPV3 with defective hair growth in rodents. *J. Invest. Dermatol.* 126 2664–2672. 10.1038/sj.jid.5700468 16858425

[B4] Bieber T. (2008). Atopic dermatitis. *N. Engl. J. Med.* 358 1483–1494. 10.1056/NEJMra074081 18385500

[B5] Bierbower S. M. Choveau F. S. Lechleiter J. D. Shapiro M. S. (2015). Augmentation of M-type (KCNQ) potassium channels as a novel strategy to reduce stroke-induced brain injury. *J. Neurosci.* 35 2101–2011. 10.1523/JNEUROSCI.3805-14.2015 25653366 PMC4315836

[B6] Biervert C. Schroeder B. C. Kubisch C. Berkovic S. F. Propping P. Jentsch T. J. (1998). A potassium channel mutation in neonatal human epilepsy. *Science* 279 403–406. 10.1126/science.279.5349.403 9430594

[B7] Bourane S. Duan B. Koch S. C. Dalet A. Britz O. Garcia-Campmany L. (2015). Gate control of mechanical itch by a subpopulation of spinal cord interneurons. *Science* 350 550–554. 10.1126/science.aac8653 26516282 PMC4700934

[B8] Brown D. A. Passmore G. M. (2009). Neural KCNQ (Kv7) channels. *Br. J. Pharmacol* 156 1185–1195. 10.1111/j.1476-5381.2009.00111.x 19298256 PMC2697739

[B9] Carey E. J. Ali A. H. Lindor K. D. (2015). Primary biliary cirrhosis. *Lancet* 386 1565–1575. 10.1016/S0140-6736(15)00154-3 26364546

[B10] Caterina M. J. Leffler A. Malmberg A. B. Martin W. J. Trafton J. Petersen-Zeitz K. R. (2000). Impaired nociception and pain sensation in mice lacking the capsaicin receptor. *Science* 288 306–313. 10.1126/science.288.5464.306 10764638

[B11] Caterina M. J. Schumacher M. A. Tominaga M. Rosen T. A. Levine J. D. Julius D. (1997). The capsaicin receptor: a heat-activated ion channel in the pain pathway. *Nature.* 389 816–824. 10.1038/39807 9349813

[B12] Cheng X. Jin J. Hu L. Shen D. Dong X. P. Samie M. A. (2010). TRP channel regulates EGFR signaling in hair morphogenesis and skin barrier formation. *Cell* 141 331–343. 10.1016/j.cell.2010.03.013 20403327 PMC2858065

[B13] Chindo B. A. Ya’U J. Danjuma N. M. Okhale S. E. Gamaniel K. S. Becker A. (2014). Behavioral and anticonvulsant effects of the standardized extract of Ficus platyphylla stem bark. *J. Ethnopharmacol.* 154 351–360. 10.1016/j.jep.2014.03.061 24754912

[B14] Crozier R. A. Ajit S. K. Kaftan E. J. Pausch M. H. (2007). MrgD activation inhibits KCNQ/M-currents and contributes to enhanced neuronal excitability. *J. Neurosci.* 27 4492–4496. 10.1523/JNEUROSCI.4932-06.2007 17442834 PMC6672314

[B15] Cruzblanca H. Koh D. S. Hille B. (1998). Bradykinin inhibits M current via phospholipase C and Ca^2+^ release from IP3-sensitive Ca^2+^ stores in rat sympathetic neurons. *Proc. Natl. Acad. Sci. U.S.A.* 95 7151–7156. 10.1073/pnas.95.12.7151 9618554 PMC22770

[B16] Cui T. T. Wang G. X. Wei N. N. Wang K. (2018). A pivotal role for the activation of TRPV3 channel in itch sensations induced by the natural skin sensitizer carvacrol. *Acta Pharmacol. Sin.* 39 331–335. 10.1038/aps.2017.152 29094727 PMC5843833

[B17] Dong X. Dong X. (2018). Peripheral and Central Mechanisms of Itch. *Neuron* 98 482–494. 10.1016/j.neuron.2018.03.023 29723501 PMC6022762

[B18] Dong X. Han S. Zylka M. J. Simon M. I. Anderson D. J. (2001). A diverse family of GPCRs expressed in specific subsets of nociceptive sensory neurons. *Cell* 106 619–632. 10.1016/s0092-8674(01)00483-4 11551509

[B19] Du X. Gao H. Jaffe D. Zhang H. Gamper N. (2018). M-type K^+^ channels in peripheral nociceptive pathways. *Br. J. Pharmacol.* 175 2158–2172. 10.1111/bph.13978 28800673 PMC5980636

[B20] Du X. Hao H. Gigout S. Huang D. Yang Y. Li L. (2014). Control of somatic membrane potential in nociceptive neurons and its implications for peripheral nociceptive transmission. *Pain* 155 2306–2322. 10.1016/j.pain.2014.08.025 25168672 PMC4247381

[B21] Dunford P. J. Williams K. N. Desai P. J. Karlsson L. McQueen D. Thurmond R. L. (2007). Histamine H4 receptor antagonists are superior to traditional antihistamines in the attenuation of experimental pruritus. *J. Allergy Clin. Immunol.* 119 176–183. 10.1016/j.jaci.2006.08.034 17208599

[B22] Elewski B. Alexis A. F. Lebwohl M. Stein Gold L. Pariser D. Del Rosso J. (2019). Itch: an under-recognized problem in psoriasis. *J. Eur. Acad. Dermatol. Venereol.* 33 1465–1476. 10.1111/jdv.15450 30680819

[B23] Ezeamuzie C. I. Igbigbi P. S. Asomugha L. Ambakederemo A. W. Abila B. Assem E. S. (1990). Urine methylhistamine concentrations before and after chloroquine in healthy black subjects. *J. Trop. Med. Hyg.* 93 423–425. 2270009

[B24] Feng J. Luo J. Yang P. Du J. Kim B. S. Hu H. (2018). Piezo2 channel-Merkel cell signaling modulates the conversion of touch to itch. *Science* 360 530–533. 10.1126/science.aar5703 29724954 PMC6114129

[B25] Gamper N. Shapiro M. S. (2003). Calmodulin mediates Ca^2+^-dependent modulation of M-type K^+^ channels. *J. Gen. Physiol.* 122 17–31. 10.1085/jgp.200208783 12810850 PMC2234471

[B26] Gamper N. Shapiro M. S. (2015). “KCNQ Channels,” in *Handbook of Ion Channels*, 1st Edn, eds Zheng J. Trudeau M. C. (Boca Raton, FL: CRC Press), 275–306. 10.1201/b18027-24

[B27] Green A. D. Young K. K. Lehto S. G. Smith S. B. Mogil J. S. (2006). Influence of genotype, dose and sex on pruritogen-induced scratching behavior in the mouse. *Pain* 124 50–58. 10.1016/j.pain.2006.03.023 16697529

[B28] Han L. Dong X. (2014). Itch mechanisms and circuits. *Annu. Rev. Biophys.* 43 331–355. 10.1146/annurev-biophys-051013-022826 24819620 PMC4081479

[B29] Jentsch T. J. (2000). Neuronal KCNQ potassium channels: physiology and role in disease. *Nat. Rev. Neurosci.* 1 21–30. 10.1038/35036198 11252765

[B30] Jia Z. Bei J. Rodat-Despoix L. Liu B. Jia Q. Delmas P. (2008). NGF inhibits M/KCNQ currents and selectively alters neuronal excitability in subsets of sympathetic neurons depending on their M/KCNQ current background. *J. Gen. Physiol.* 131 575–587. 10.1085/jgp.200709924 18474635 PMC2391251

[B31] King C. H. Lancaster E. Salomon D. Peles E. Scherer S. S. (2014). Kv7.2 regulates the function of peripheral sensory neurons. *J. Comp. Neurol.* 522 3262–3280. 10.1002/cne.23595 24687876 PMC4428907

[B32] Kosenko A. Hoshi N. (2013). A change in configuration of the calmodulin-KCNQ channel complex underlies Ca^2+^-dependent modulation of KCNQ channel activity. *PLoS One* 8:e82290. 10.1371/journal.pone.0082290 24349250 PMC3857245

[B33] Lai-Cheong J. E. Sethuraman G. Ramam M. Stone K. Simpson M. A. McGrath J. A. (2012). Recurrent heterozygous missense mutation, p.Gly573Ser, in the TRPV3 gene in an Indian boy with sporadic Olmsted syndrome. *Br. J. Dermatol.* 167 440–442. 10.1111/j.1365-2133.2012.11115.x 22835024

[B34] Lin Z. Chen Q. Lee M. Cao X. Zhang J. Ma D. (2012). Exome sequencing reveals mutations in TRPV3 as a cause of Olmsted syndrome. *Am. J. Hum. Genet.* 90 558–564. 10.1016/j.ajhg.2012.02.006 22405088 PMC3309189

[B35] Linley J. E. Rose K. Patil M. Robertson B. Akopian A. N. Gamper N. (2008). Inhibition of M current in sensory neurons by exogenous proteases: a signaling pathway mediating inflammatory nociception. *J. Neurosci.* 28 11240–11249. 10.1523/JNEUROSCI.2297-08.2008 18971466 PMC6087463

[B36] Liu B. Liang H. Liu L. Zhang H. (2008). Phosphatidylinositol 4,5-bisphosphate hydrolysis mediates histamine-induced KCNQ/M current inhibition. *Am. J. Physiol. Cell Physiol.* 295 C81–C91. 10.1152/ajpcell.00028.2008 18448631

[B37] Liu B. Linley J. E. Du X. Zhang X. Ooi L. Zhang H. (2010). The acute nociceptive signals induced by bradykinin in rat sensory neurons are mediated by inhibition of M-type K^+^ channels and activation of Ca^2+^-activated Cl^–^ channels. *J. Clin. Invest.* 120 1240–1252. 10.1172/JCI41084 20335661 PMC2846053

[B38] Liu Q. Sikand P. Ma C. Tang Z. Han L. Li Z. (2012). Mechanisms of itch evoked by b-alanine. *J. Neurosci.* 32 14532–14537. 10.1523/JNEUROSCI.3509-12.2012 23077038 PMC3491570

[B39] Liu Q. Tang Z. Surdenikova L. Kim S. Patel K. N. Kim A. (2009). Sensory neuron-specific GPCR Mrgprs are itch receptors mediating chloroquine-induced pruritus. *Cell* 139 1353–1365. 10.1016/j.cell.2009.11.034 20004959 PMC2989405

[B40] Meixiong J. Dong X. (2017). Mas-related G protein-coupled receptors and the biology of itch sensation. *Annu. Rev. Genet.* 51 103–121. 10.1146/annurev-genet-120116-024723 29178819

[B41] Morita T. McClain S. P. Batia L. M. Pellegrino M. Wilson S. R. Kienzler M. A. (2015). HTR7 mediates serotonergic acute and chronic itch. *Neuron* 87 124–138. 10.1016/j.neuron.2015.05.044 26074006 PMC4536073

[B42] Mucha M. Ooi L. Linley J. E. Mordaka P. Dalle C. Robertson B. (2010). Transcriptional control of KCNQ channel genes and the regulation of neuronal excitability. *J. Neurosci.* 30 13235–13245. 10.1523/JNEUROSCI.1981-10.2010 20926649 PMC3044881

[B43] Nilius B. Szallasi A. (2014). Transient receptor potential channels as drug targets: from the science of basic research to the art of medicine. *Pharmacol. Rev.* 66 676–814. 10.1124/pr.113.008268 24951385

[B44] Passmore G. M. Selyanko A. A. Mistry M. Al-Qatari M. Marsh S. J. Matthews E. A. (2003). KCNQ/M currents in sensory neurons: significance for pain therapy. *J. Neurosci.* 23 7227–7236. 10.1523/JNEUROSCI.23-18-07227 12904483 PMC6740665

[B45] Peier A. M. Reeve A. J. Andersson D. A. Moqrich A. Earley T. J. Hergarden A. C. (2002). A heat-sensitive TRP channel expressed in keratinocytes. *Science* 296 2046–2049. 10.1126/science.1073140 12016205

[B46] Pereira M. P. Ständer S. (2018). Therapy for pruritus in the elderly: a review of treatment developments. *Expert Opin. Pharmacother.* 19 443–450. 10.1080/14656566.2018.1444752 29493371

[B47] Rau K. K. McIlwrath S. L. Wang H. Lawson J. J. Jankowski M. P. Zylka M. J. (2009). Mrgprd enhances excitability in specific populations of cutaneous murine polymodal nociceptors. *J. Neurosci.* 29 8612–8619. 10.1523/JNEUROSCI.1057-09.2009 19571152 PMC2756673

[B48] Rose K. Ooi L. Dalle C. Robertson B. Wood I. C. Gamper N. (2011). Transcriptional repression of the M channel subunit Kv7.2 in chronic nerve injury. *Pain* 152 742–754. 10.1016/j.pain.2010.12.028 21345591 PMC3071978

[B49] Shim W. S. Tak M. H. Lee M. H. Kim M. Kim M. Koo J. Y. (2007). TRPV1 mediates histamine-induced itching via the activation of phospholipase A2 and 12-lipoxygenase. *J. Neurosci.* 27 2331–2337. 10.1523/JNEUROSCI.4643-06.2007 17329430 PMC6673467

[B50] Shimada S. G. LaMotte R. H. (2008). Behavioral differentiation between itch and pain in mouse. *Pain* 139 681–687. 10.1016/j.pain.2008.08.002 18789837 PMC2723192

[B51] Sikand P. Shimada S. G. Green B. G. LaMotte R. H. (2009). Similar itch and nociceptive sensations evoked by punctate cutaneous application of capsaicin, histamine and cowhage. *Pain* 144 66–75. 10.1016/j.pain.2009.03.001 19423224 PMC2694489

[B52] Sikand P. Shimada S. G. Green B. G. LaMotte R. H. (2011). Sensory responses to injection and punctate application of capsaicin and histamine to the skin. *Pain* 152 2485–2494. 10.1016/j.pain.2011.06.001 21802851 PMC3199342

[B53] Simons F. E. Simons K. J. (2011). Histamine and H1-antihistamines: celebrating a century of progress. *J. Allergy Clin. Immunol.* 128 1139–1150.e4. 10.1016/j.jaci.2011.09.005 22035879

[B54] Simonsen E. Komenda P. Lerner B. Askin N. Bohm C. Shaw J. (2017). Treatment of uremic pruritus: a systematic review. *Am. J. Kidney Dis.* 70 638–655. 10.1053/j.ajkd.2017.05.018 28720208

[B55] Singh N. A. Charlier C. Stauffer D. DuPont B. R. Leach R. J. Melis R. (1998). A novel potassium channel gene. KCNQ2, is mutated in an inherited epilepsy of newborns. *Nat. Genet.* 18 25–29. 10.1038/ng0198-25 9425895

[B56] Spradley J. M. Davoodi A. Carstens M. I. Carstens E. (2012). Opioid modulation of facial itch- and pain-related responses and grooming behavior in rats. *Acta Derm. Venereol.* 92 515–520. 10.2340/00015555-1364 22513524

[B57] Sun Y. G. Chen Z. F. (2007). A gastrin-releasing peptide receptormediates the itch sensation in the spinal cord. *Nature* 448 700–703. 10.1038/nature06029 17653196

[B58] Tatulian L. Delmas P. Abogadie F. C. Brown D. A. (2001). Activation of expressed KCNQ potassium currents and native neuronal M-type potassium currents by the anti-convulsant drug retigabine. *J. Neurosci.* 21 5535–5545. 10.1523/JNEUROSCI.21-15-05535.2001 11466425 PMC6762632

[B59] Than J. Y. Li L. Hasan R. Zhang X. (2013). Excitation and modulation of TRPA1, TRPV1, and TRPM8 channel-expressing sensory neurons by the pruritogen chloroquine. *J. Biol. Chem.* 288 12818–12827. 10.1074/jbc.M113.450072 23508958 PMC3642326

[B60] Wang G. Wang K. (2017). The Ca^2+^-permeable cation transient receptor potential TRPV3 channel: An emerging pivotal target for itch and skin diseases. *Mol. Pharmacol.* 92 193–200. 10.1124/mol.116.107946 28377424

[B61] Wang H. S. Pan Z. Shi W. Brown B. S. Wymore R. S. Cohen I. S. (1998). KCNQ2 and KCNQ3 potassium channel subunits: molecular correlates of the M-channel. *Science* 282 1890–1893. 10.1126/science.282.5395.1890 9836639

[B62] Wilson S. R. Gerhold K. A. Bifolck-Fisher A. Liu Q. Patel K. N. Dong X. (2011). TRPA1 is required for histamine-independent, Mas-related G protein-coupled receptor-mediated itch. *Nat. Neurosci.* 14 595–602. 10.1038/nn.2789 21460831 PMC3181150

[B63] Yamamoto-Kasai E. Imura K. Yasui K. Shichijou M. Oshima I. Hirasawa T. (2012). TRPV3 as a therapeutic target for itch. *J. Invest. Dermatol.* 132 2109–2112. 10.1038/jid.2012.97 22475759

[B64] Yoshioka T. Imura K. Asakawa M. Suzuki M. Oshima I. Hirasawa T. (2009). Impact of the Gly573Ser substitution in TRPV3 on the development of allergic and pruritic dermatitis in mice. *J. Invest. Dermatol.* 129 714–722. 10.1038/jid.2008.245 18754035

[B65] Young M. A. Thomas S. A. (2014). M1-muscarinic receptors promote fear memory consolidation via phospholipase C and the M-current. *J. Neurosci.* 34 1570–1578. 10.1523/JNEUROSCI.1040-13.2014 24478341 PMC3905134

[B66] Zhang F. Gigout S. Liu Y. Wang Y. Hao H. Buckley N. J. (2019). Repressor element 1-silencing transcription factor drives the development of chronic pain states. *Pain* 160 2398–2408. 10.1097/j.pain.0000000000001633 31206463 PMC6756259

[B67] Zhang X. Zhang H. Zhou N. Xu J. Si M. Jia Z. (2015). Tannic acid modulates excitability of sensory neurons and nociceptive behavior and the Ionic mechanism. *Eur. J. Pharmacol.* 764 633–642. 10.1016/j.ejphar.2015.06.048 26134502

[B68] Zhang X. F. Han P. Neelands T. R. McGaraughty S. Honore P. Surowy C. S. (2011). Coexpression and activation of TRPV1 suppress the activity of the KCNQ2/3 channel. *J. Gen. Physiol.* 138 341–352. 10.1085/jgp.201110618 21844219 PMC3171082

[B69] Zheng Q. Fang D. Liu M. Cai J. Wan Y. Han J. S. (2013). Suppression of KCNQ/M (Kv7) potassium channels in dorsal root ganglion neurons contributes to the development of bone cancer pain in a rat model. *Pain* 154 434–448. 10.1016/j.pain.2012.12.005 23352759

[B70] Zheng Y. Xu H. Zhan L. Zhou X. Chen X. Gao Z. (2015). Activation of peripheral KCNQ channels relieves gout pain. *Pain* 156 1025–1035. 10.1097/j.pain.0000000000000122 25735002 PMC4450870

