# Supplementary figures and images for: Inhibition of M/Kv7 Currents Contributes to Chloroquine-Induced Itch in Mice
Source: Front Mol Neurosci. 2020 Jun 30;13:105. doi: 10.3389/fnmol.2020.00105 (PMC7339983; doi:10.3389/fnmol.2020.00105)

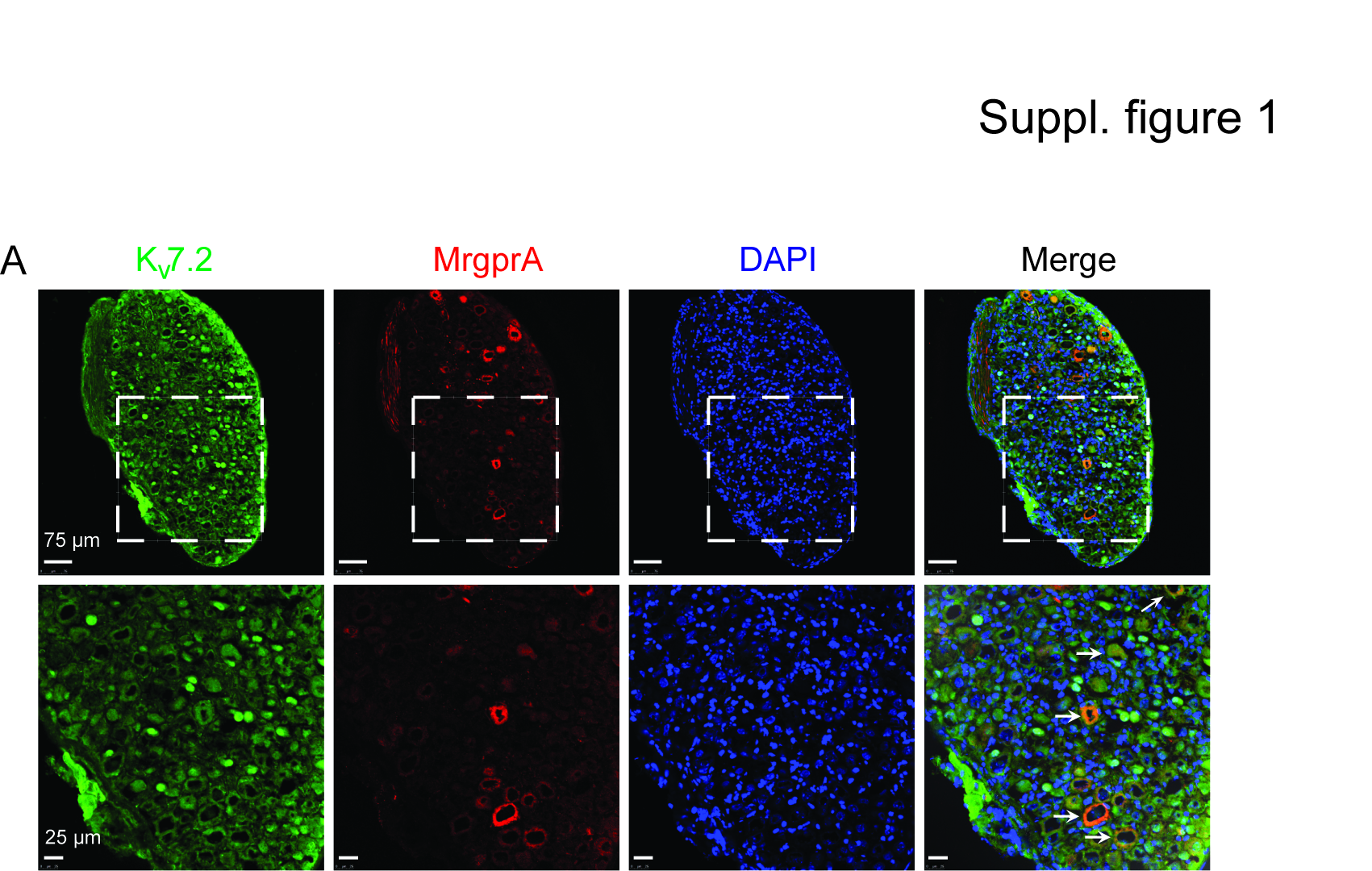

Supplement: FIGURE S1 — Co-localization of Kv7.2 and MrgprA in mouse DRG neurons. (A) Representative images show that M/Kv7 channel subunit Kv7.2 is co-localized with MrgprA receptor (arrows, bottom merged) in mouse DRG neurons. Images in the bottom row are extended from the square regions in the up row./break Scale bar in each panel in up and bottom row is 75 and 25 μm, respectively. [file Image_1.TIF]
